# A study of the effects of synthesis conditions on Li_5_FeO_4_/carbon nanotube composites

**DOI:** 10.1038/srep46530

**Published:** 2017-04-19

**Authors:** Suk-Woo Lee, Hyun-Kyung Kim, Myeong-Seong Kim, Kwang Chul Roh, Kwang-Bum Kim

**Affiliations:** 1 Department of Material Science and Engineering, Yonsei University, 134 Shinchon-dong, Seodaemoon-gu, Seoul, 120-749, Republic of Korea; 2 Department of Materials Science and Metallurgy, University of Cambridge, 27 Charles Babbage Road, Cambridge CB3 0FS, UK; 3 Energy Efficient Materials Team, Energy & Environmental Division, Korea Institute of Ceramic Engineering & Technology, 101 Soho-ro, Jinju-si, Gyeongsangnam-do, 660-031, Republic of Korea

## Abstract

Li_5_FeO_4_/carbon nanotube (LFO/CNT) composites composed of sub-micron sized LFO and a nanocarbon with high electrical conductivity were successfully synthesized for the use as lithium ion predoping source in lithium ion cells. The phase of LFO in the composite was found to be very sensitive to the synthesis conditions, such as the heat treatment temperature, type of lithium salt, and physical state of the precursors (powder or pellet), due to the carbothermic reduction of Fe_3_O_4_ by CNTs during high temperature solid state reaction. Under optimized synthesis conditions, LFO/CNT composites could be synthesized without the formation of impurities. To the best of our knowledge, this is the first report on the synthesis and characterization of a sub-micron sized LFO/CNT composites.

The Fe-based oxide Li_5_FeO_4_ (LFO), which has an antifluorite-type structure, Li_5_V_2_FeO_4_ (V: vacancy), has a considerably high specific charge capacity (~700 mAh g^−1^) when 4 mol of Li^+^ ions are extracted electrochemically^1^. However, it is accompanied by a severe irreversible structural disruption when Li^+^ ions are extracted from the host material. Therefore, LFO becomes electrochemically inactive after the first charge process^1^,^2^.

However, there are a few reports on the use of LFO as a cathode material in lithium ion batteries (LIB). For example, Narukawa *et al*. analyzed the structural changes of LFO as increasing number of Li^+^ ions electrochemically extracted from and inserted into the host. It was shown that a 0.5 equivalent Li^+^ ion could be deintercalated from and intercalated into the host LFO without irreversible structural changes, and LFO was suggested as a potential cathode material^3^.

It should be noted, however, that LFO should not be considered as a cathode material for LIBs. LFO has a discharge potential of around 2.5 V, which is considerably lower than that of conventional cathode materials (i.e., LiCoO_2_ (3.7 V), LiMn_2_O_4_ (4.0 V), LiFePO_4_ (3.4 V))^3^,^4^,^5^. In addition, under the general lower cut-off voltage of cathode materials (3 V), the specific capacity of LFO is as low as 25 mAh g^−1^ 3,4,5. Furthermore, the cyclability of LFO is very poor. For example, Okumura *et al*. reported that the capacity of LFO at the 10^th^ cycle was 73% of that measured at the 3^rd^ cycle^5^. The low specific capacity and poor cyclability of LFO are hardly acceptable for cathode materials in LIB applications.

Recently, LFO has been studied as a lithium ion predoping source in lithium ion cells^1^,^2^. According to these reports^1^,^2^, lithium ion predoping sources should meet the following requirements. First, they should possess a large number of available Li^+^ ions in the unit structure to provide a sufficient number of Li^+^ ions to anode materials during electrochemical charging. Second, the Li^+^ ions extracted from the host lithium ion predoping sources should not be allowed to return to its former structure after the first charging process, which means that the candidate materials must have a high electrochemical irreversibility after the first cycle. Considering the two requirements of a lithium ion predoping source in lithium ion cells, LFO could be a very effective lithium ion predoping source as LFO provides a large number of Li^+^ ions with a capacity of ~700 mAh g^−1^ during the first charge, and the extracted Li^+^ cannot reversibly return to its initial structure during the subsequent discharge.

However, for LFO to be a more effective lithium ion predoping source, the low electrical conductivity of LFO, which is due to the disconnection of FeO_4_^5−^ tetrahedra from each other in the structure^6^, should be improved. In addition, LFO is usually prepared through a solid-state reaction at high temperature (>800 °C) over 72 h^2^. As a result of the harsh heat treatment conditions, the particle size of LFO tends to be over several tens of micrometers, which is obviously not suitable for lithium ion predoping sources. The low electrical conductivity and large particle size of LFO could limit the power density of lithium ion cells, as the charged LFO particles remaining in the cathode become insulating, resulting in an increase in the resistivity of lithium ion cells^2^. Therefore, the synthesis of sub-micron sized LFO with high electrical conductivity is still a challenge in applying LFO as an effective lithium ion predoping source.

Our approach to these issues associated with LFO is to employ a hybrid composite composed of sub-micron sized LFO and a nanocarbon with high electrical conductivity. The dispersion of LFO in a highly conductive nanocarbon frame hinders the growth and agglomeration of the oxide particles and improves electrical conductivity^7^,^8^,^9^,^10^,^11^,^12^,^13^,^14^.

In this study, we report the synthesis of LFO/carbon nanotube (LFO/CNT) composites by a simple solid state method using a Fe_3_O_4_/CNT nanocomposite and lithium salts as precursors under tightly controlled synthesis conditions. The heat treatment temperature, type of lithium salts used, and physical states of the precursors (powder or pellet) were carefully controlled to achieve successful synthesis LFO/CNT composites without impurities. To the best of our knowledge, this is the first report on the synthesis and characterization of LFO/CNT composites.

## Results and Discussion

### Fe_3_O_4_/CNT nanocomposite

**Figure 1:**
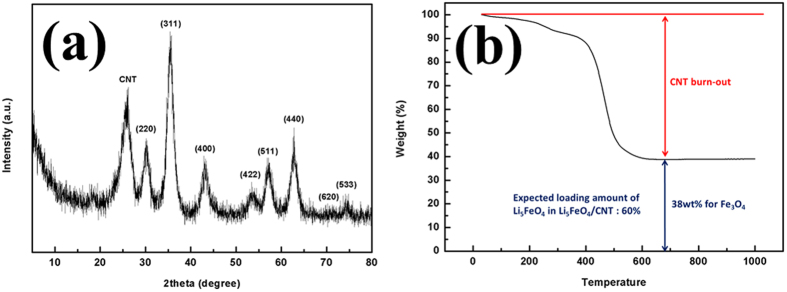
(**a**) XRD pattern and (**b**) TGA curve of the Fe_3_O_4_/CNT nanocomposite obtained in an air atmosphere.

Figure 1(a) shows the X-ray diffraction (XRD) pattern of the Fe_3_O_4_/CNT nanocomposite. The nanocomposite exhibits an inverse-spinel Fe_3_O_4_ formation, which is consistent with previous reports^15^. A strong peak attributable to the carbon phase (CNT) in the Fe_3_O_4_/CNT nanocomposite is observed in the XRD pattern. Thermogravimetric analysis (TGA) was carried out to analyze the loading amount of Fe_3_O_4_ in the nanocomposite. Figure 1(b) shows the TGA curve of the Fe_3_O_4_/CNT nanocomposite. The TGA data indicate that the prepared Fe_3_O_4_/CNT nanocomposite contains 62 wt% carbon. On the basis of the TGA results, the amount of carbon in the LFO/CNT composite was estimated to be 40 wt%.

**Figure 2:**
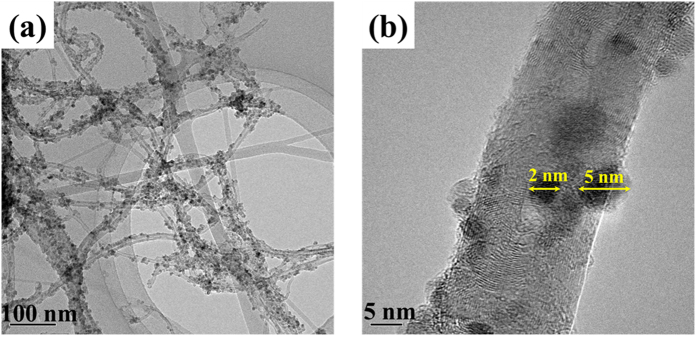
TEM images of the Fe_3_O_4_/CNT nanocomposite at (**a**) low magnification and (**b**) high magnification.

The particle size of Fe_3_O_4_ is one of the crucial factors to determine the particle size of LFO in the LFO/CNT composite because the particle size of LFO is proportional to that of Fe_3_O_4_. (see Supporting Information Figure S1). To synthesize LFO with sub-micron size, it is necessary to synthesize nano-sized Fe_3_O_4_. Figure 2 shows the field-emission transmission electron microscopy (FE-TEM) images of the Fe_3_O_4_/CNT nanocomposite. It is clearly observable that the Fe_3_O_4_ nanoparticles are uniformly distributed over the CNT surface in the nanocomposite. It is also evident that the nanocomposite did not have any separate Fe_3_O_4_ nanoparticles disconnected from the CNTs. In addition, the particle size of the Fe_3_O_4_ nanoparticles was estimated to be as small as 2~5 nm, which has the advantage of reducing the particle size of LFO in the LFO/CNT composite.

### Synthesis of LFO/CNT composites

The phase of LFO in the composite was found to be very sensitive to the synthesis conditions, such as the heat treatment temperature, type of lithium salts, and physical state of the precursors (powder or pellet), owing to the carbothermic reduction of Fe_3_O_4_ by the CNTs during the high temperature solid state reaction. Therefore, we carefully controlled the heat treatment temperature, type of lithium salts, and states of the precursors (powder or pellet) to successfully synthesize LFO/CNT composites. The details of the composites and their synthesis conditions are summarized in Table 1.

### Heat treatment temperature

**Figure 3:**
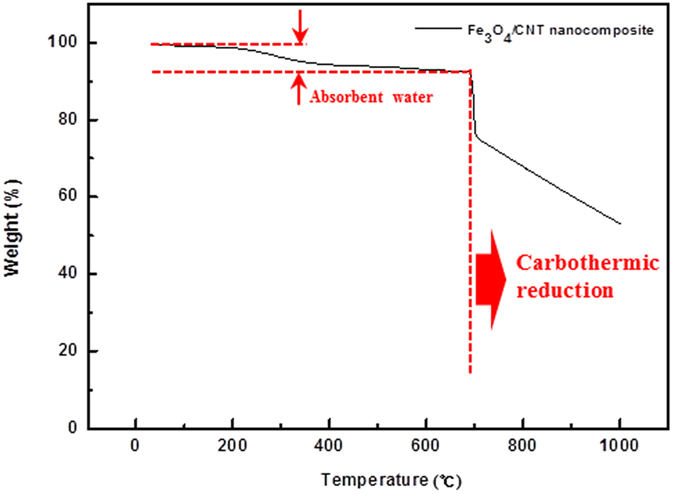
TGA curve of the Fe_3_O_4_/CNT nanocomposite obtained in a nitrogen atmosphere.

In order to determine the heat treatment temperature for the synthesis of LFO/CNT composites, TGA of a Fe_3_O_4_/CNT nanocomposite in a nitrogen atmosphere was carried out. Figure 3 shows the TGA curve of the Fe_3_O_4_/CNT nanocomposite obtained in nitrogen atmosphere. The weight loss of 5–6% at the initial stage could be attributed to the evaporation of absorbed water in the Fe_3_O_4_/CNT nanocomposite. As the temperature was increased to 700 °C, no further weight loss was observed. However, a significant weight loss was observed at temperatures above 700 °C, which was due to the carbothermic reduction of Fe_3_O_4_ by the CNTs.

A carbothermic reduction is the chemical reaction between metal oxides and carbon that forms metal and CO or CO_2_ under an inert atmosphere. In the case presented here, Fe_3_O_4_ reacts with carbon and forms metallic Fe and CO or CO_2_, which can be expressed as follows^16^,^17^,^18^:


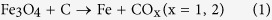


In other words, above 700 °C, carbothermic reduction of Fe_3_O_4_ by CNTs can occur which causes an oxygen deficiency for the synthesis of LFO. Therefore, this reaction should be avoided for the successful synthesis of LFO. Based on the TGA analysis, the upper limit of the heat treatment temperature was set to 700 °C in the synthesis of LFO/CNT composites.

**Figure 4:**
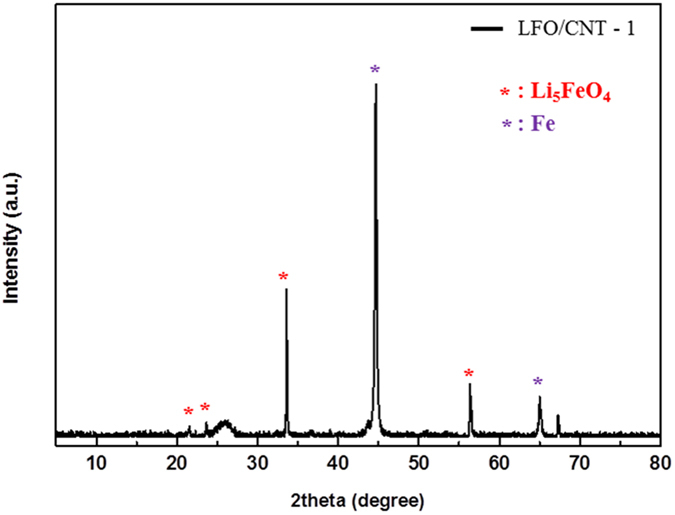
XRD pattern of LFO/CNT-1.

To investigate the effect of the heat treatment temperature on the synthesis of LFO/CNT composites, we mixed Li_2_O and the Fe_3_O_4_/CNT nanocomposite as precursors and heat-treated the mixture at various temperatures. Figure 4 shows the XRD pattern of LFO/CNT-1. When the heat treatment temperature was 700 °C (onset temperature of the carbothermic reduction reaction), the XRD patterns can be indexed mostly to metallic Fe due to the carbothermic reduction of Fe_3_O_4_ by the CNTs, as indicated in Fig. 3.

**Figure 5:**
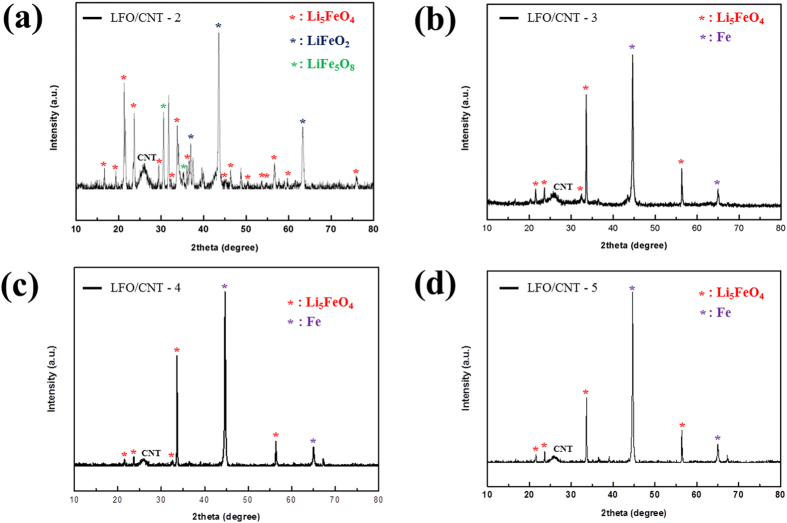
XRD patterns of (**a**) LFO/CNT-2, (**b**) LFO/CNT-3, (**c**) LFO/CNT-4, and (**d**) LFO/CNT-5.

In order to suppress the carbothermic reduction, the heat treatment temperature was set to 630, 635, 640, and 650 °C for LFO/CNT-2, LFO/CNT-3, LFO/CNT-4, and LFO/CNT-5 samples, respectively. Since the carbothermic reduction reaction is critically dependent on the temperature, the heat treatment temperature was controlled in an interval of 5–10 °C. The corresponding XRD patterns are shown in Fig. 5. At 630 °C, LFO peaks (indicated by red asterisks) were detected. However, numerous impurity phases, such as LiFeO_2_ (blue asterisks) and LiFe_5_O_8_ (green asterisks), were also detected. Interestingly, no metallic Fe peaks were detected, which suggests the absence of the carbothermic reduction of Fe_3_O_4_ at 630 °C. As the heat treatment temperature was gradually increased to 635, 640, and 650 °C, metallic Fe peaks began to appear in the XRD patterns of the LFO/CNT-3, LFO/CNT-4, and LFO/CNT-5 samples, which means that carbothermic reduction has occurred. As shown in Fig. 3, the onset temperature of the carbothermic reduction reaction is around 700 °C. Therefore, the origin of the formation of metallic Fe in LFO/CNT-3, LFO/CNT-4, and LFO/CNT-5 samples in spite of the heat treatment temperature below 700 °C should be discussed.

It was reported that Li_2_O can be reduced to gaseous Li by the carbothermic reduction reaction as follows^19^:


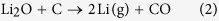


The reduction of Li_2_O to gaseous Li results in the loss of Li. Consequently, only the Fe_3_O_4_/CNT nanocomposite exists in the system. Careful analysis of the carbothermic reduction reactions reported in the literature reveals that progress of the carbothermic reduction is dependent not only on the heat treatment temperature but also on the heat treatment time^17^,^18^. Therefore, the carbothermic reduction can occur in spite of low heat treatment temperatures because the heat treatment is continued for a long period of time (72 h).

Based on the XRD results described above, heat treatment temperatures of 630, 635, and 640 °C were selected to synthesize LFO/CNT composites.

### Lithium salts

As demonstrated above, Li_2_O is not an appropriate lithium salt to synthesize LFO/CNT composites. Li_2_O is reduced to gaseous Li, resulting in the formation of metallic Fe as impurity. Therefore, other types of lithium salts, such as Li_2_O_2_, LiOH, LiNO_3_, and Li_2_CO_3_ should be considered as the lithium source. Among them, Li_2_O_2_, LiOH, and LiNO_3_ decompose thermally to Li_2_O at temperatures below 600 °C^20^,^21^. Therefore, we chose Li_2_CO_3_ as the lithium source because it does not decompose thermally to Li_2_O in the temperature range of 630–640 °C (see Supporting Information Figure S2)^22^.

**Figure 6:**
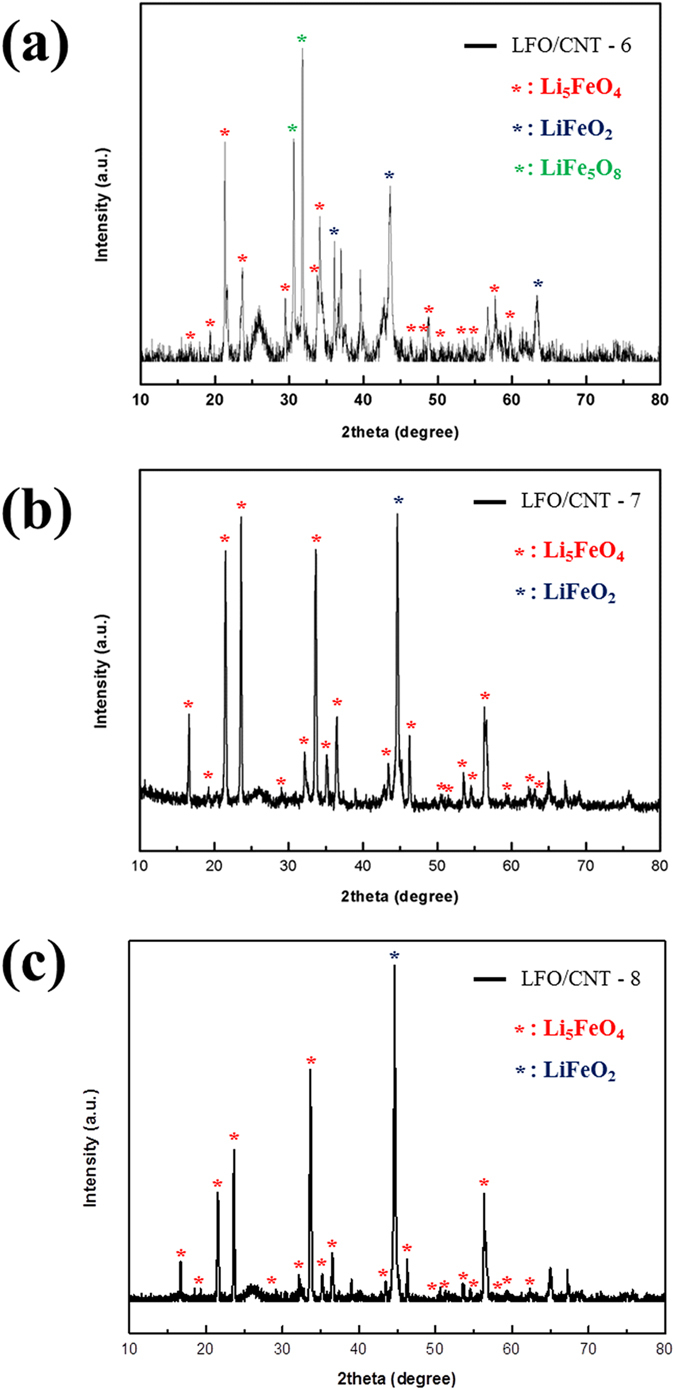
XRD patterns of (**a**) LFO/CNT-6, (**b**) LFO/CNT-7, and (**c**) LFO/CNT-8.

Figure 6(a–c) show the XRD patterns of the developed LFO/CNT-6, LFO/CNT-7, and LFO/CNT-8 composites, respectively. The XRD pattern of the LFO/CNT-6 composite is similar to that of the LFO/CNT-2 composite. At 630 °C, not only LFO peaks (red asterisks) but also numerous impurity phases, such as LiFeO_2_ (blue asterisks) and LiFe_5_O_8_ (green asterisks), were detected. Metallic Fe peaks were not detected. Meanwhile, the XRD results of LFO/CNT-7 and LFO/CNT-8 were considerably different from those of LFO/CNT-3 and LFO/CNT-4. Although the heat treatment temperatures were the same (635 and 640 °C, respectively), metallic Fe peaks were not detected in the LFO/CNT-7 and LFO/CNT-8 composites. In addition, LFO peaks were clearly detected, which means that Li_2_CO_3_ reacted with the Fe_3_O_4_/CNT nanocomposite without inducing a carbothermic reduction reaction. However, in the XRD patterns of LFO/CNT-7 and LFO/CNT-8, LiFeO_2_ peaks were observed, and the intensities of the LiFeO_2_ peaks were stronger at higher heat treatment temperatures. Based on these results, we chose 635 °C as the heat treatment temperature and Li_2_CO_3_ as the lithium salt.

### Physical state of precursor (powder or pellet)

**Figure 7:**
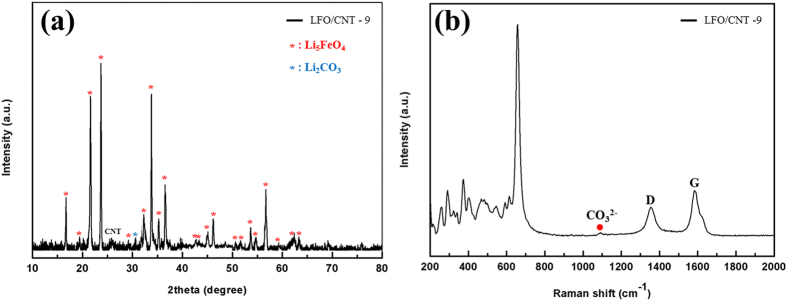
(**a**) XRD patterns and (**b**) Raman spectrum of LFO/CNT-9.

According to previous reports, using pellets for heat treatment offer the advantage of synthesizing various compounds with even composition because pellets provide a larger contact area between precursors for solid state reactions than powders^23^. To investigate the effect of the physical state of the precursors, we fabricated pellets from a mixture of the Fe_3_O_4_/CNT nanocomposite and Li_2_CO_3_ by compression at a high pressure. After the pellets were fabricated, they were heat treated under the same synthesis conditions as those of LFO/CNT-7. Figure 7(a) shows the XRD pattern of LFO/CNT-9. As mentioned above, the LFO/CNT-7 composite exhibited strong LiFeO_2_ impurity peaks. However, no LiFeO_2_ peak was detected for the LFO/CNT-9 sample that was synthesized using pellets because the increased contact area between Li_2_CO_3_ and the Fe_3_O_4_/CNT nanocomposite leads to a more uniform solid state reaction^23^. Finally, all XRD peaks of LFO/CNT-9 can be indexed to LFO except for one minor peak of Li_2_CO_3_ between 30° and 32° originating from the sensitivity of LFO to CO_2_ in air^1^.

A Raman analysis was conducted to investigate the phase of the LFO/CNT-9 composite further. Figure 7(b) shows the Raman spectrum of LFO/CNT-9. In this spectrum, all of the peaks in the wavelength range of 200–800 cm^−1^ are indexed to LFO, which is consistent with the results previously reported for LFO materials^24^. The Raman spectrum of the LFO/CNT composite also contained two peaks at ~1349 cm^−1^ and ~1580 cm^−1^, which correspond to the D and G bands of the CNTs, respectively. The G band corresponds to the first-order scattering of the E_2g_ phonon of the sp^2^ carbon domains in a two-dimensional hexagonal lattice, and the D band corresponds to the disorder-induced mode associated with structural defects and imperfections^25^,^26^. The Raman spectrum of the LFO/CNT composite also contains a Li_2_CO_3_ peak at ~1090 cm^−1^, which is consistent with the XRD results.

We also conducted an X-ray photoelectron spectroscopy (XPS) analysis to demonstrate the successful synthesis of the LFO/CNT composite. Figure S3 shows the Fe 2p spectrum of the LFO/CNT-9 composite. The typical Fe 2p spectrum is split into 2p_3/2_ and 2p_1/2_ peaks, and the 2p_3/2_ peak at 711.2 eV corresponds to Fe^3+ ^27,28. In Figure S3, the Fe 2p_3/2_ peak is detected at 711.2 eV, which means that the oxidation state of Fe is +3. The oxidation state of Fe in LFO/CNT-9 is consistent with previous reports^2^,^5^,^29^.

Inductively coupled plasma optical emission spectroscopy (ICP/OES) was used to determine the precise elemental composition of the LFO/CNT-9 sample. The composition ratio of Li to Fe in LFO/CNT-9 was 5.2:1, which is similar to the starting composition ratio of Li to Fe (see Supporting Information Table S1).

As shown in Fig. 1, we were able to determine the amount of carbon in the LFO/CNT composite as approximately 40 wt%, on the basis of the assumption that there was no carbon loss due to carbothermic reduction reaction. We carried out an elemental analysis (EA) to determine the amount of carbon in the LFO/CNT-9 sample and found it to be 33.2 wt%. This suggests that no severe carbothermic reduction reaction occurred under the optimized synthesis conditions (see Supporting Information Table S2).

**Figure 8:**
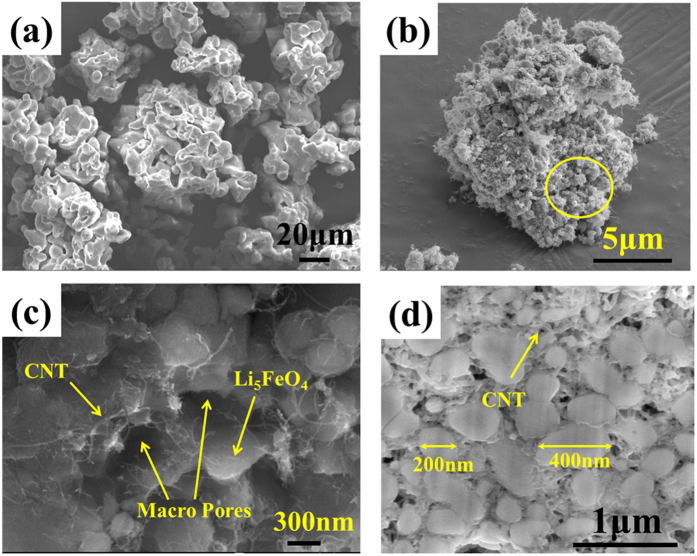
SEM images of (**a**) bare LFO, (**b**) LFO/CNT-9 at a low magnification, (**c**) LFO/CNT-9 at a high magnification, and (**d**) a cross section of LFO/CNT-9.

Figure 8(a) shows a field emission scanning electron microscopy (FE-SEM) image of bare LFO particles. The particle size of bare LFO is larger than several tens of micrometers due to the high heat treatment temperature of 900 °C and the long heat treatment time of 72 h. Figure 8(b,c) show FE-SEM images of the LFO/CNT-9 composite. The images show that the composite is composed of LFO particles and CNTs with a uniform distribution. To confirm the uniform dispersion of the LFO particles and CNTs inside the secondary particles, cross-sectional SEM observation of the LFO/CNT-9 composite sample was performed using a focused ion beam (FIB) (Fig. 8(d)). The results show that CNT and LFO particles are evenly dispersed without agglomeration. In addition, the particle size of LFO in the composite was measured to 200–400 nm, which is much smaller than that of bare LFO, as a nano sized Fe_3_O_4_/CNT nanocomposite was used as precursor, as shown in Fig. 2.

A TEM analysis was also carried out to confirm the morphology of the LFO/CNT-9 material. Figure S4(a) and (b) show TEM images of the LFO/CNT-9 material. The particle size of the LFO particles is about 200–400 nm, and CNTs were observed between these LFO particles. The TEM results demonstrate a uniform distribution of CNTs and LFO particles, which is consistent with the SEM analysis.

Table 2 summarizes the electrical conductivities of the bare LFO and LFO/CNT-9 samples, which is obtained using a two-point probe method with the DC voltage sweep method. Bare LFO is found to be an insulator. Meanwhile, the conductivity of LFO/CNT-9 is 1.71 S cm^−1^, which is much higher than that of bare LFO owing to the uniform dispersion of CNT in the composite. (see Supporting Information Figure S5).

By combining SEM, TEM, and electrical conductivity results of the LFO/CNT-9 composite, it is concluded that the LFO/CNT composite synthesized in this study could be a promising lithium ion predoping source candidate due to its sub-micron particle size and high electrical conductivity.

## Conclusion

A LFO/CNT composite was successfully synthesized to be used as a lithium ion predoping source for the first time using a simple solid state method. Antifluorite-structured LFO/CNT composites were obtained under a tightly controlled synthesis condition. In particular, heat treatment temperature and lithium salt are carefully controlled to suppress the carbothermic reduction of Fe_3_O_4_ by CNTs. Additionally, impurity formations are effectively inhibited by applying pellets as precursors for heat treatment.

The final LFO/CNT product obtained under the optimized synthesis conditions has a particle size of 200–400 nm, which is much smaller than that of bare LFO. Furthermore, the electrical conductivity was remarkably increased to 1.71 S cm^−1^ since the CNTs provide continuous electron pathways in the LFO/CNT composite.

Due to the small particle size and high electrical conductivity properties, the LFO/CNT composite can be considered an effective lithium ion predoping source.

## Methods

### Synthesis of the Fe_3_O_4_/CNT nanocomposite as precursor

The details of the synthesis method were described in our previous report^15^. Briefly, acid-treated CNTs (0.1 g) were sonicated in diethylene glycol (DEG, >99%, Fluka, 80 mL), after which FeCl_2_ ∙ 4H_2_O (0.062 g, Aldrich), FeCl_3_ ∙ 6H_2_O (0.087 g, Junsei), and ammonium hydroxide (15 ml, 30 wt%, Junsei) were added to the solution. The solution was then loaded into 100 ml Teflon vessels that were sealed and placed in a microwave system (MARS-5, CEM Corporation). The reaction mixture was heated to 200 °C and maintained at that temperature for 10 min.

### Synthesis of LFO/CNT composites

LFO/CNT composites were prepared by a solid state reaction using the prepared Fe_3_O_4_/CNT nanocomposite and various lithium salts as starting materials. The molar ratio of lithium to iron was 5.5:1. Because Fe_3_O_4_ is not very reactive and lithium evaporates before the reaction is complete, excess lithium salts are used to obtain a single phase^29^. After mixing the precursors in a ball milling machine, the precursor powders or pellets were fired at various temperatures under flowing argon for 72 h. For the purpose of comparison, a bare LFO material was also prepared using a solid state heat treatment method at a temperature of 900 °C for 72 h. Both final products were handled carefully and stored in the glove box because of their structural instability with respect to H_2_O and CO_2_.

### Characterization

TGA data for Fe_3_O_4_/CNT nanocomposites were collected on a thermal analysis instrument (TGA/DSC 1, Mettler Toledo) with a heating rate of 10 °C min^−1^ in an air or nitrogen flow rate of 50 mL min^−1^. XRD (Rigaku, Cu Kα, 40 kV, 20 mA) patterns were obtained at room temperature in the 2θ range of 10°–80° in intervals of 4° min^−1^. Raman spectroscopy (T64000, Jobin-Yvon) was carried out to confirm the phase of the LFO in each LFO/CNT composite. XPS measurements were conducted to measure the oxidation state of Fe using a Thermo MultiLab 2000 spectrometer with a monochromatic Al K_α_ X-ray source. The binding energy for the spectrum was calibrated using the C 1 s peak (285 eV) as a reference. An EA (2400 Series II, Perkin Elmer) was carried out to measure the amount of carbon in the final products. The molar ratio of Li to Fe in each LFO/CNT composite was measured by ICP/OES (OPTIMA 8300, PerkinElmer). The morphology of the as-prepared samples was characterized using TEM (CM200, Philips) and SEM (JEOL-7800F, JEOL, Ltd.). To investigate the internal structures of the samples, cross-sectional SEM images were obtained using a focused ion beam (FIB) (JIB-4601F, JEOL, Ltd.). The electrical conductivity was measured using a two-point probe method with the DC voltage sweep method (VMP3, Bio-Logic), as described in detail in our previous study^30^.

## Additional Information

**How to cite this article**: Lee, S.-W. *et al*. A study of the effects of synthesis conditions on Li_5_FeO_4_/carbon nanotube composites. *Sci. Rep.* **7**, 46530; doi: 10.1038/srep46530 (2017).

**Publisher's note:** Springer Nature remains neutral with regard to jurisdictional claims in published maps and institutional affiliations.

## Supplementary Material

Supporting Information

## Figures and Tables

**Table 1:** Summary of the sample preparation conditions for the LFO/CNT composites developed in this study.

Composites	Heat treatment temperature	Reactants	Precursor state
LFO/CNT-1	700 °C	Li_2_O + Fe_3_O_4_/CNT	powder
LFO/CNT-2	630 °C	Li_2_O + Fe_3_O_4_/CNT	powder
LFO/CNT-3	635 °C	Li_2_O + Fe_3_O_4_/CNT	powder
LFO/CNT-4	640 °C	Li_2_O + Fe_3_O_4_/CNT	powder
LFO/CNT-5	650 °C	Li_2_O + Fe_3_O_4_/CNT	powder
LFO/CNT-6	630 °C	Li_2_CO_3_ + Fe_3_O_4_/CNT	powder
LFO/CNT-7	635 °C	Li_2_CO_3_ + Fe_3_O_4_/CNT	powder
LFO/CNT-8	640 °C	Li_2_CO_3_ + Fe_3_O_4_/CNT	powder
LFO/CNT-9	635 °C	Li_2_CO_3_ + Fe_3_O_4_/CNT	pellet

**Table 2:** Electrical conductivities of the bare LFO and LFO/CNT-9 samples.

Sample name	Electrical conductivity (S cm^−1^)
Bare LFO	~0 (insulator)
LFO/CNT-9	1.71
