# Supplementary material for: A study of the effects of synthesis conditions on Li5FeO4/carbon nanotube composites
Source: Sci Rep. 2017 Apr 19;7:46530. doi: 10.1038/srep46530 (PMC5396188; doi:10.1038/srep46530)
Supplement: Supporting Information [file srep46530-s1.doc]

A study of the effects of synthesis conditions on Li_5_FeO_4_/carbon nanotube composites

Suk-Woo Lee^a^, Hyun-Kyung Kim^a,b^, Myeong-Seong Kim^a^, Kwang Chul Roh^c*^ and Kwang-Bum Kim^a*^

^a^Department of Material Science and Engineering, Yonsei University, 134 Shinchon-dong, Seodaemoon-gu, Seoul 120-749, Republic of Korea

^b^Department of Materials Science and Metallurgy, University of Cambridge, 27 Charles Babbage Road, Cambridge CB3 0FS, UK

^c^Energy Efficient Materials Team, Energy & Environmental Division, Korea Institute of Ceramic Engineering & Technology, 101 Soho-ro, Jinju-si, Gyeongsangnam-do, 660-031, Republic of Korea

**Electrochemical tests of the LFO/CNT composites**

Electrochemical tests were performed at room temperature in a glove box using a three-electrode cell with two Li metal foils as counter and reference electrodes. The working electrode was prepared by mixing the LFO/CNT (90 wt%) composite with polyvinylidene fluoride (10 wt%, PVDF; Aldrich), dissolved in N-methylpyrrolidone (NMP; Aldrich), as a binder. The resulting slurry was coated onto a titanium foil and then dried. The amount of slurry used to form the electrode was approximately 1–2 mg cm^-2^. A potentiostat/galvanostat (MPG2, Bio-logic) was used for the galvanostatic charge–discharge tests and cyclic voltammetry tests. The cut-off voltages for the charge/discharge tests were 2.6–4.5 V versus Li/Li^+^. The electrolyte consisted of 1 M LiPF_6_ dissolved in a mixture of ethyl carbonate (EC) and dimethyl carbonate (DMC) with a volume ratio of 1:1.

**Supplementary Figures**

**
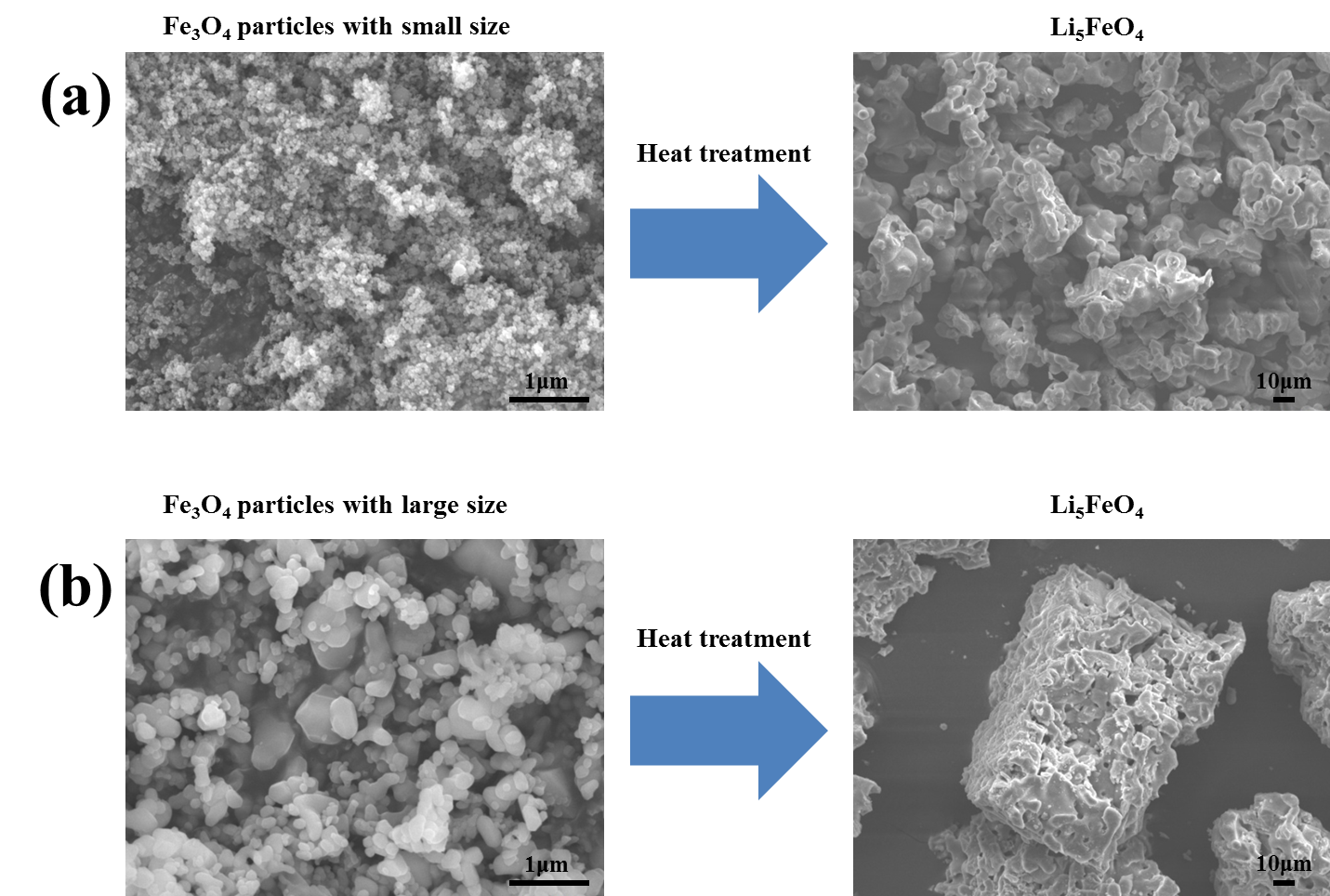
**

**Figure S1.** Relation between the particle size of Fe_3_O_4_ and that of LFO

**
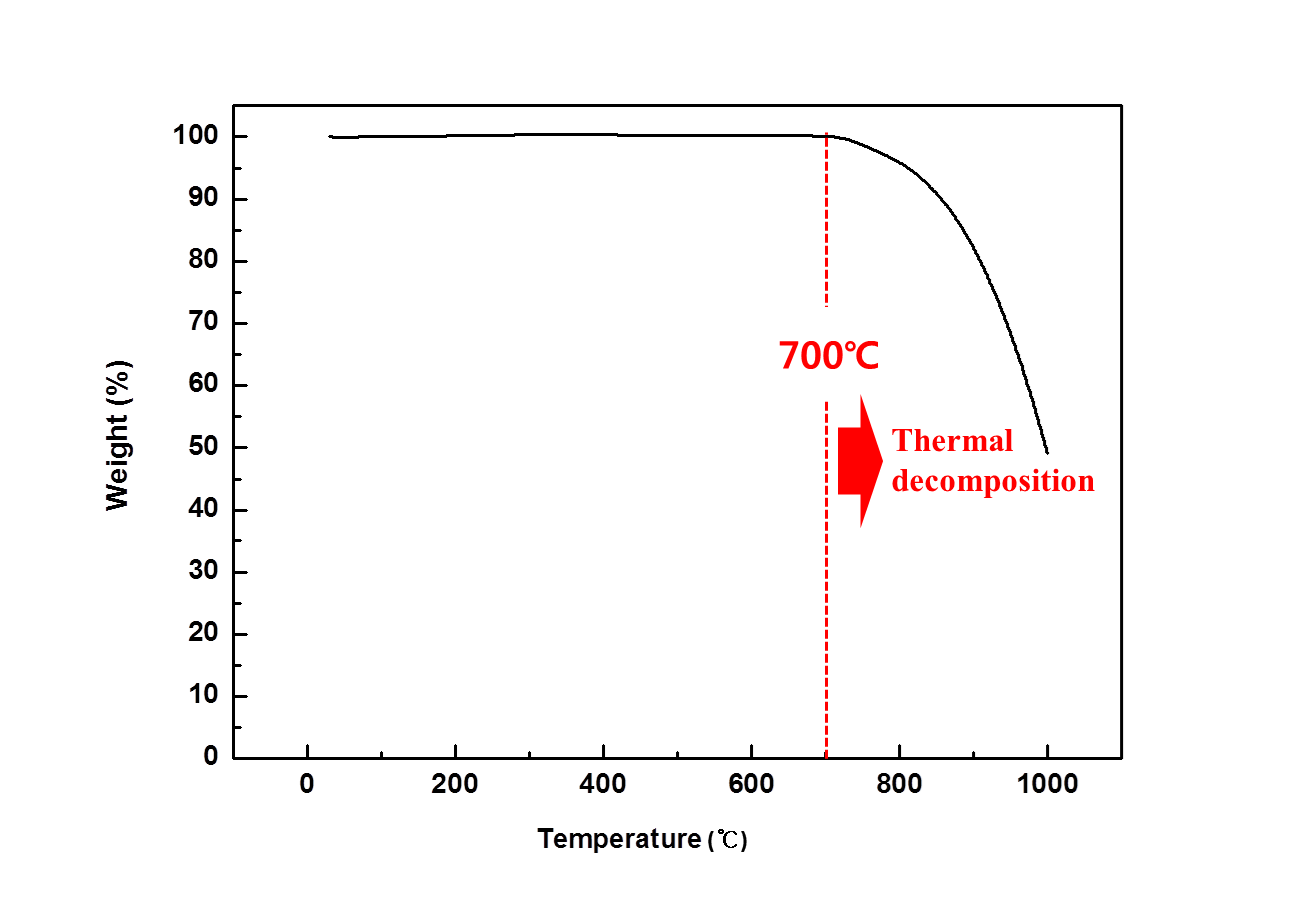
**

**Figure S2.** TGA curve of Li_2_CO_3_ obtained in nitrogen atmosphere


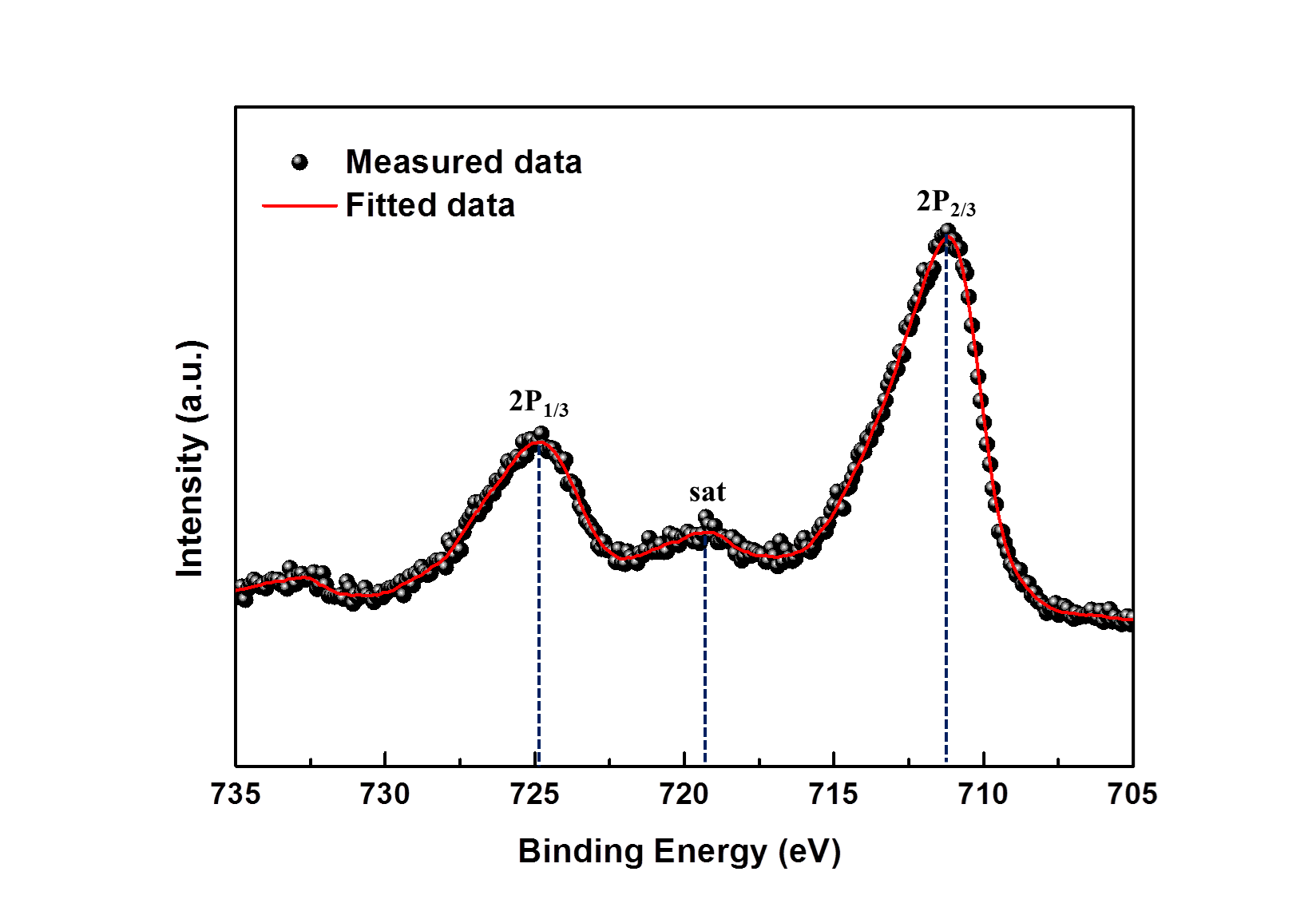


**Figure S3.** XPS spectrum for Fe_2p_ of the LFO/CNT-9 sample

**
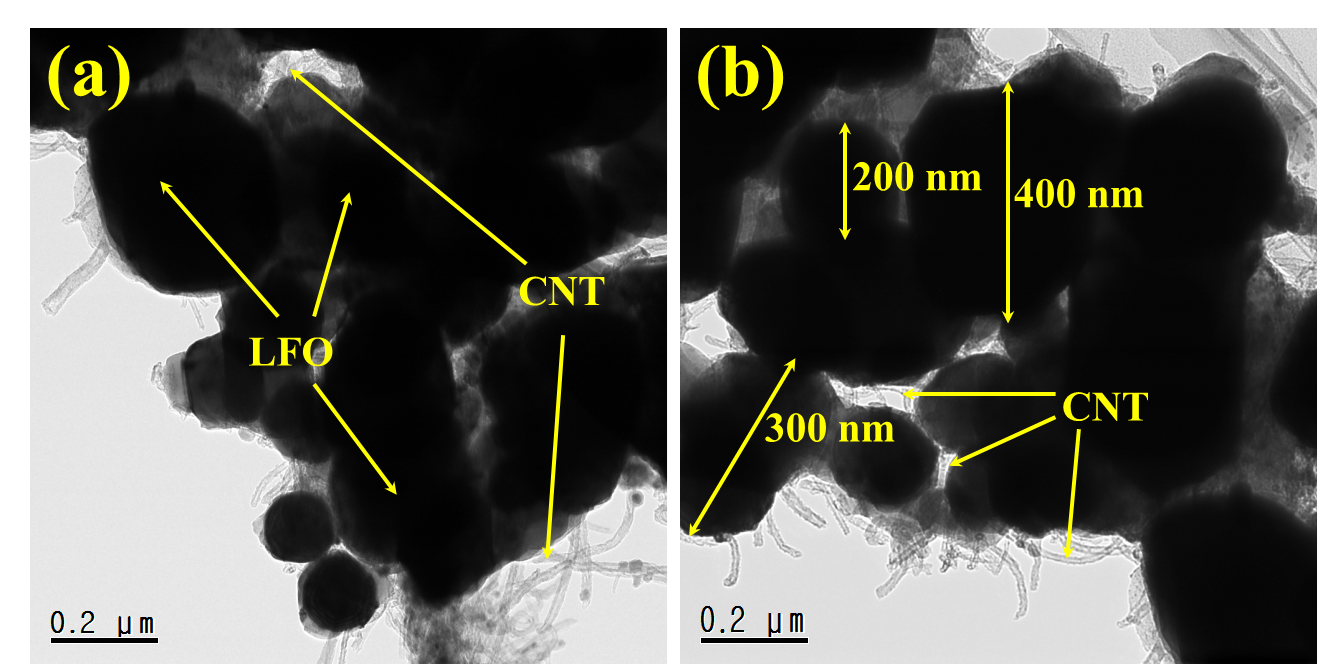
**

**Figure S4.** (a, b) TEM images of the LFO/CNT-9 sample


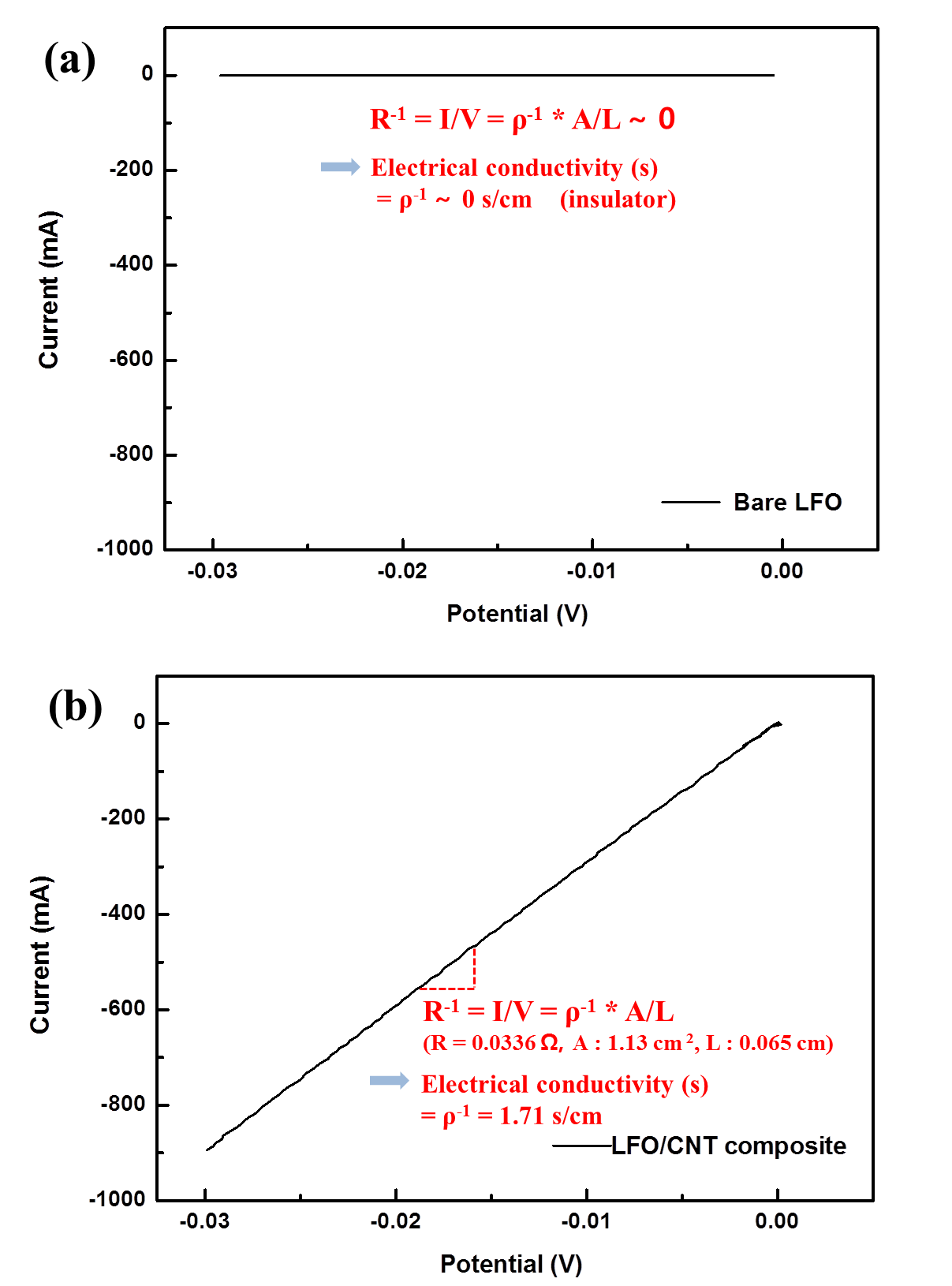


**Figure S5.** I-V curves of bare LFO and LFO/CNT-9 samples


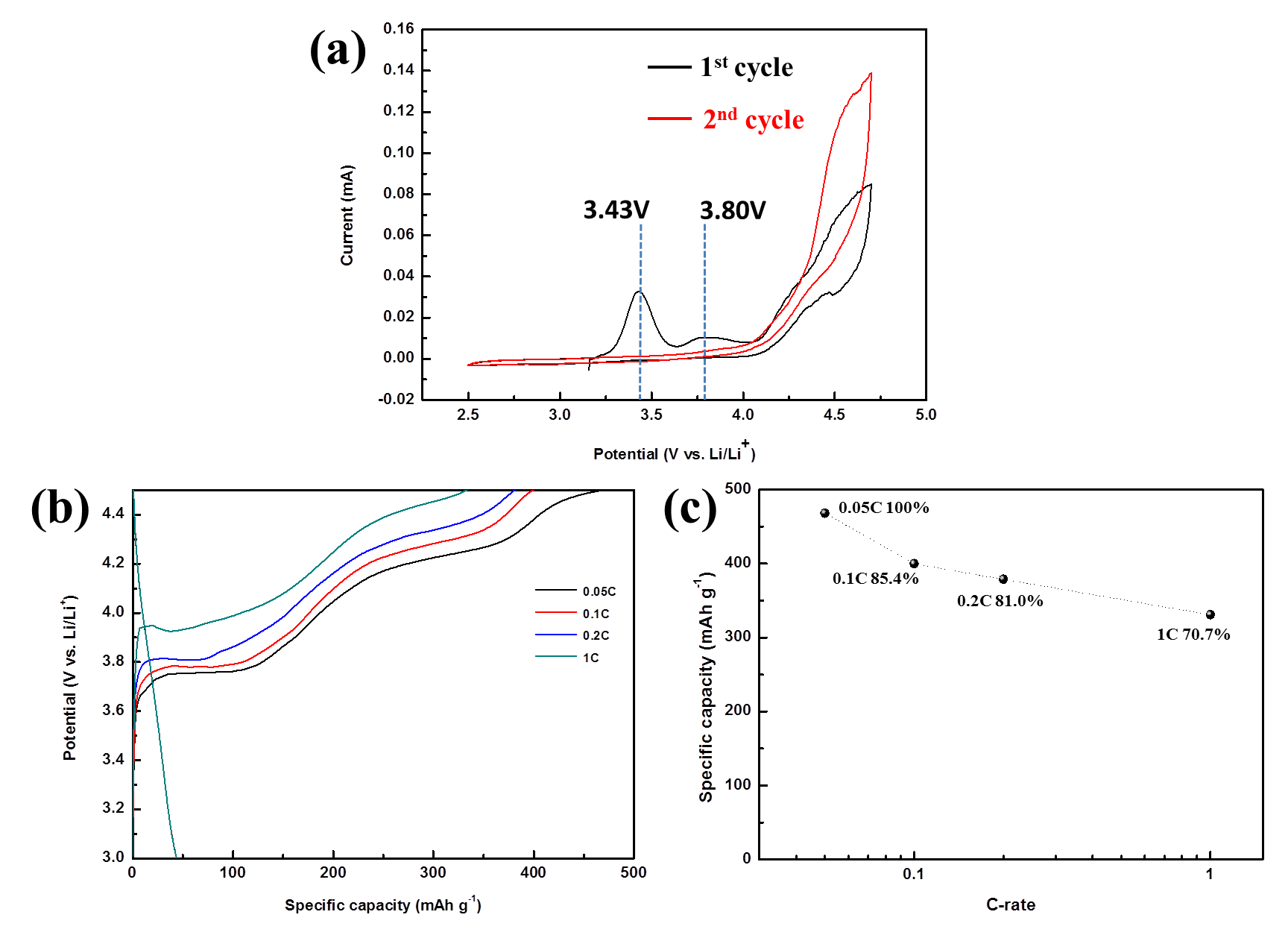


**Figure S6.** (a) Cyclic voltammograms (CVs) of the LFO/CNT-9 sample at a scan rate of 0.05 mV s^-1^, (b) Charge–discharge curves at various C-rates, and (c) the rate capability of the LFO/CNT-9 sample.

We measured the electrochemical performance of the LFO/CNT composites as lithium ion predoping sources to confirm the hybrid effect with CNTs. Figure S6(a) shows the CVs of the LFO/CNT-9 sample measured at a scan rate of 0.05 mV s^-1^. The black and red lines indicate the 1^st^ and 2^nd^ cycles, respectively. In the 1^st^ cycle, two clear anodic peaks are observed at 3.43 and 3.80 V. In the 2^nd^ cycle, the anodic peaks disappeared, which means that irreversible structural changes were caused by the deintercalation of Li^+^ ions,^1^ which is the typical electrochemical behavior of LFO. Figure S6(b) shows the charge–discharge curves and Figure S6(c) shows the rate capability of the LFO/CNT composites. Each charge/discharge curve was obtained in a fresh cell since LFO is only electrochemically active during the first charge. In the charge curves, two clear plateaus for LFO are observed. The charge capacity of the LFO/CNT composite was measured to be 468, 400, 379, and 331 mAh g^−1^ at 0.05, 0.1, 0.2, and 1 C-rate.

The LFO/CNT composite undergoes a severe phase transition during the fabrication of a coin cell due to the poor chemical stability of LFO by H_2_O and CO_2_ in the atmosphere, resulting in a relatively low specific discharge capacity at a low C-rate.^2^ (It is difficult to avoid contact with air completely because small amounts of H_2_O and CO­_2_ always exist in a dry room. In particular, for some processes, we could not isolate the materials from air, such as slurry casting on the substrate and the drying processes.) In spite of the phase transition, the LFO/CNT composite shows good electrochemical performance until 1 C-rate (30–40 times higher current densities than previous reports) due to the increased electrical conductivity by the CNT network and reduced particle size.

**Table S1.** ICP/OES analysis results for LFO/CNT-9

| Sample name | Li | Fe |
| --- | --- | --- |
| LFO/CNT-9 | 5.2 | 1 |

**Table S2.** Elemental analysis (EA) results for LFO/CNT-9

|  | Results (wt%) | | | | |
| --- | --- | --- | --- | --- | --- |
|  | Carbon | Hydrogen | Oxygen | Nitrogen | Sulfur |
| LFO/CNT-9 | 33.2 | 1.5 | 28.7 | - | - |

**Reference**

1 Park, M. S. *et al.* Scalable Integration of Li5FeO4 towards Robust, High-Performance Lithium-Ion Hybrid Capacitors. *Chemsuschem* **7**, 3138-3144, doi:10.1002/cssc.201402397 (2014).

2 Johnson, C. S. *et al.* Li2O Removal from Li5FeO4: A Cathode Precursor for Lithium-Ion Batteries. *Chem Mater* **22**, 1263-1270, doi:10.1021/cm902713m (2010).
